# Clinical Profile of RSV Breakthrough Infections After Nirsevimab Introduction

**DOI:** 10.3390/v18101107

**Published:** 2026-10-07

**Authors:** Anna Chiara Vittucci, Livia Antilici, Alessandra Salvatori, Domenico Umberto De Rose, Rossana Scutari, Velia Chiara Di Maio, Lelia Rotondi Aufiero, Mara Pisani, Anna Maria Musolino, Andrea Dotta, Carlo Federico Perno, Alberto Villani

**Affiliations:** 1 General Pediatric and Infectious Disease Unit, Pediatric Emergency Medicine, Bambino Gesù Children’s Hospital, IRCCS, 00165 Rome, Italy; annachiara.vittucci@opbg.net (A.C.V.); alessandra.salvatori@opbg.net (A.S.); lelia.rotondiaufiero@opbg.net (L.R.A.); mara.pisani@opbg.net (M.P.); amcaterina.musolino@opbg.net (A.M.M.); alberto.villani@opbg.net (A.V.); 2 Neonatal Intensive Care Unit, Bambino Gesù Children’s Hospital, IRCCS, 00165 Rome, Italy; domenico.derose@opbg.net (D.U.D.R.); andrea.dotta@opbg.net (A.D.); 3 Microbiology and Diagnostic Immunology Unit, Bambino Gesù Children’s Hospital, IRCCS, 00165 Rome, Italy; rossana.scutari@opbg.net (R.S.); veliachiara.dimaio@opbg.net (V.C.D.M.); carlofederico.perno@opbg.net (C.F.P.); 4 Systems Medicine Departments, Tor Vergata University of Rome, 00133 Rome, Italy

**Keywords:** RSV, bronchiolitis, nirsevimab, breakthrough infections, immunoprophylaxis, universal prophylaxis

## Abstract

The introduction of universal prophylaxis with nirsevimab for all children in their first epidemic season has substantially reduced the burden of respiratory syncytial virus (RSV). However, a small number of breakthrough infections continue to occur despite immunization, mostly in previously healthy infants, and these cases remain incompletely characterized. We conducted a retrospective study across three epidemic seasons at a third-level hospital, including 536 RSV-positive children under 12 months of age hospitalised for bronchiolitis. Immunised infants with breakthrough infection showed a higher prevalence of prematurity and comorbidities than unimmunised infants, and were more frequently represented in the 9–12-month age group, with a median of 291 days since nirsevimab administration. Immunised infants had significantly lower odds of requiring supplemental oxygen (aOR 0.33, 95% CI 0.19–0.57, *p* < 0.001). However, once oxygen therapy was required, immunisation status did not independently predict the need for advanced ventilatory support. Severe breakthrough cases requiring invasive mechanical ventilation occurred even among infants without traditional risk factors. These findings confirm the substantial real-world impact of universal nirsevimab prophylaxis while underscoring the need for continued clinical vigilance and further research into the mechanisms underlying breakthrough disease.

## 1. Introduction

Respiratory syncytial virus (RSV) is a negative-sense, single-stranded RNA virus belonging to the Pneumoviridae family, and it is classified into two main subtypes, A and B, which are defined by differences in the G protein [1]. RSV-A and RSV-B often co-circulate during the same epidemic season, with alternating geographic or annual predominance [2].

RSV is the leading cause of acute lower respiratory tract infections (LRTIs) in children worldwide [3,4]. The burden is particularly high among infants under 6 months of age, who account for more than 38% of RSV-related LRTI admissions and 50% of deaths under 5 years of age [5]. Although several well-known risk factors for severe disease have been identified, such as prematurity and cardiopulmonary comorbidities [6,7], most bronchiolitis cases occur in otherwise healthy infants [8,9].

For this reason, significant efforts have been made in recent decades to develop pharmacological prevention methods that could be administered to the entire pediatric population during their first RSV epidemic season, and to high-risk infants up until their second epidemic season, rather than just to high-risk infants, as was the case with palivizumab [6,10,11,12,13]. Nirsevimab, a long-acting monoclonal antibody that targets the site Ø of the prefusion form of RSV F protein, was introduced into the infant immunisation schedule in Europe in autumn 2023, with significant differences among countries [14,15]. Italy introduced nirsevimab in the 2024–25 epidemic season, with a heterogeneous schedule among regions due to its extensive regional autonomy [16,17]. It was only in the 2025–26 epidemic season that the distribution among birth cohorts became uniform across Italy [18,19].

Introducing universal prophylaxis against RSV for all children in their first epidemic season has produced outstanding results, with an effectiveness rate of 82–83% in preventing hospitalisation and 67–69% in preventing critical care for RSV-LRTI in infants [20,21]. Real-world data indicate greater uncertainty regarding nirsevimab effectiveness among selected high-risk groups, such as preterm infants and children with congenital heart disease [22,23], although definitive conclusions on effect modification by prematurity or comorbidities are still limited [14].

However, a small number of breakthrough infections continue to occur despite immunisation. Most reported cases occur in previously healthy infants, and the clinical severity of these cases remains incompletely characterised [23,24,25,26,27]. In fact, among the small proportion of infants hospitalised with RSV despite prophylaxis, evidence on disease severity compared with hospitalised unimmunised infants is still limited and inconsistent, with some studies showing reduced respiratory support [23,28] and others showing no clear differences in in-hospital outcomes [26,29,30].

Recent evidence suggests that RSV may evade immune responses through mechanisms such as intercellular spread via nanotubes and syncytia, which are resistant to neutralizing antibodies, contributing to breakthrough infection despite high neutralizing titers [31].

In addition, real-world surveillance of cases across two consecutive RSV seasons in France has provided interesting molecular insights into nirsevimab breakthrough infections. French surveillance revealed a non-significant trend towards greater severity in breakthrough infections during the RSV-A-dominant 2023–24 season, and nirsevimab resistance was rare during this period (2/24 RSV-B isolates) [25]. However, during the RSV-B-dominant 2024–25 season, resistance became more prevalent (12.5% of RSV-B breakthroughs), and breakthrough infections exhibited lower intensive care unit (ICU) admission rates [32].

All these findings emphasise that neither viral resistance nor the severity of breakthrough cases follow a simple, uniform pattern, and highlight the need for detailed clinical characterisation of the small group of infants who develop severe breakthrough cases despite immunoprophylaxis. Against this background, the primary aim of this study was to describe the clinical profile of RSV breakthrough infections in hospitalised infants with bronchiolitis. Secondary analyses aimed to compare breakthrough infections with those of unimmunised infants and the link between immunisation and disease severity.

## 2. Materials and Methods

This was a monocentric, observational, retrospective study conducted at the General Pediatric and Infectious Disease Unit, Pediatric Emergency Medicine, and the Neonatal Intensive Care Unit of the Bambino Gesù Children’s Hospital, IRCCS, Rome, Italy. We retrospectively identified all patients under the age of one year who were hospitalised with a diagnosis of acute bronchiolitis and a nasopharyngeal aspirate or swab that tested positive for RSV within 72 h of admission. All infants hospitalised for bronchiolitis underwent testing with an extended multiplex PCR respiratory panel at admission, except for 6 infants who were tested with RSV-specific PCR only. Respiratory specimens were processed according to the standardized protocols of the Microbiology and Virology Laboratory. RSV positivity was defined as the detection of the virus in any respiratory specimen using either the Xpert Xpress CoV-2/Flu/RSV plus test (Cepheid, Sunnyvale, CA, USA), a multiplex real-time reverse transcription polymerase chain reaction (RT-PCR) assay for the simultaneous detection and differentiation of RSV, influenza A, influenza B, and SARS-CoV-2, or the BioFire FilmArray Respiratory Panel 2.1 Plus (bioMérieux, Marcy-l’Étoile, France), a multiplex nested PCR system for the detection of multiple respiratory pathogens. Both assays were performed according to the manufacturers’ instructions. The Xpert Xpress CoV-2/Flu/RSV plus test was performed on the GeneXpert platform using 300 μL of specimen loaded directly into single-use cartridges containing the RT-PCR reagents and enabling the automated RT-PCR process. The workflow includes sample preparation, RNA extraction, reverse transcription, and real-time PCR. Each cartridge includes two internal controls to ensure test validity. The BioFire FilmArray Respiratory Panel 2.1 Plus integrates nucleic acid extraction, nested multiplex PCR, and automated result interpretation, providing results within approximately 45 min from 300 μL of specimen. The panel detects 19 respiratory viruses and four bacteria, including RSV; influenza A (A/H1, A/H1-2009, and A/H3); influenza B; parainfluenza viruses 1–4; adenovirus; rhinovirus/enterovirus; human metapneumovirus; human coronaviruses 229E, HKU1, NL63, and OC43; SARS-CoV-2; MERS-CoV; Bordetella parapertussis; Bordetella pertussis; Chlamydia pneumoniae; and Mycoplasma pneumoniae. Each test includes two internal controls that are automatically assessed by the system to ensure test validity. Testing criteria and laboratory procedures remained consistent throughout all three epidemic seasons, ensuring a standardized approach to RSV diagnosis throughout the study period.

The study spanned three consecutive epidemic seasons, from 1 October 2023 to 31 May 2026, with the first season preceding the introduction of nirsevimab. Part of the data from the 2023–2024 and 2024–2025 seasons was included in a previous brief report focused on the impact of the regional immunisation campaign on hospital admissions [16]. The characterisation of breakthrough infections, the 2025–2026 season, the Neonatal Intensive Care Unit data and the multivariable analyses are reported here for the first time.

Patients were considered immunised if they had received nirsevimab at least 96 h before the onset of symptoms [29,33,34] and 7 days before admission.

In our cohort, comorbidities included congenital heart disease, congenital lung disease and bronchopulmonary dysplasia, genetic syndromes and other severe airway/neuromuscular conditions impairing secretion clearance. Prematurity was defined as birth before 37 completed weeks of gestation.

The severity of RSV infection was categorised based on the need for respiratory support and, when applicable, the maximum level of support received by each patient. Levels of respiratory support included: low-flow oxygen (LFO), high-flow nasal cannula (HFNC), helmet continuous positive airway pressure or non-invasive ventilation (HcPAP/NIV), and invasive mechanical ventilation (IMV). Criteria for initiating each level of respiratory support followed the hospital bronchiolitis protocol (Table S2).

Categorical variables are presented as n (%) and were compared using the chi-squared test or Fisher’s exact test where appropriate. Continuous variables are presented as the median (interquartile range) and were compared using the Kruskal–Wallis test due to their non-normal distribution. Bonferroni-corrected pairwise post-hoc comparisons were performed for significant results. Multivariable logistic regression models were used to assess the independent association between nirsevimab immunisation and the study outcomes, adjusting for potential confounders. Covariates were selected a priori, based on their established association with RSV severity in the literature (young age, prematurity and comorbidities) [3,6,7,35,36]. Because nirsevimab was available only from the 2024–2025 season, a sensitivity analysis additionally including epidemic season (2023–2024 as reference) was performed (complete model in Table S3).

A two-tailed *p*-value < 0.05 was considered statistically significant. Statistical analyses were performed using SPSS version 26.0.

## 3. Results

### 3.1. Hospitalisations for RSV Bronchiolitis

A total of 952 patients were admitted with acute bronchiolitis during the study period. Of these, 402 were admitted during the 2023–2024 epidemic season, 305 during the 2024–2025 season and 245 during the 2025–2026 season. In the 2025–2026 epidemic season only 81 patients tested positive for RSV (33.1%), a significantly lower percentage compared to the previous seasons (2023–2024: 65.9%, 2024–2025: 61.6%; *p* < 0.001). The characteristics of patients hospitalised for RSV bronchiolitis are summarised in Table 1.

### 3.2. Breakthrough Infections

In the last season, the proportion of patients hospitalised with all-cause bronchiolitis with documented nirsevimab administration was higher (80.2%), compared to the suboptimal level reached in the 2024–2025 season (24.9%, *p* < 0.001).

During the 2024–2025 epidemic season, 18 hospitalised patients with RSV bronchiolitis had received nirsevimab immunisation prior to admission, while 170 had not. During the 2025–2026 epidemic season, 63 hospitalised patients with RSV bronchiolitis had previously received nirsevimab immunisation, while 17 had not. One patient had no data available regarding nirsevimab administration. A total of 81 breakthrough infections were recorded across the two epidemic seasons.

Preterm birth was significantly more prevalent in the breakthrough infections group compared to the unimmunized group (12.3% and 5.5% respectively, see Table 2). These characteristics were also evident in the subgroup of patients who experienced a severe course of infection requiring oxygen therapy. Data are shown in Table 2 and Supplementary Table S1.

**Figure 1:**
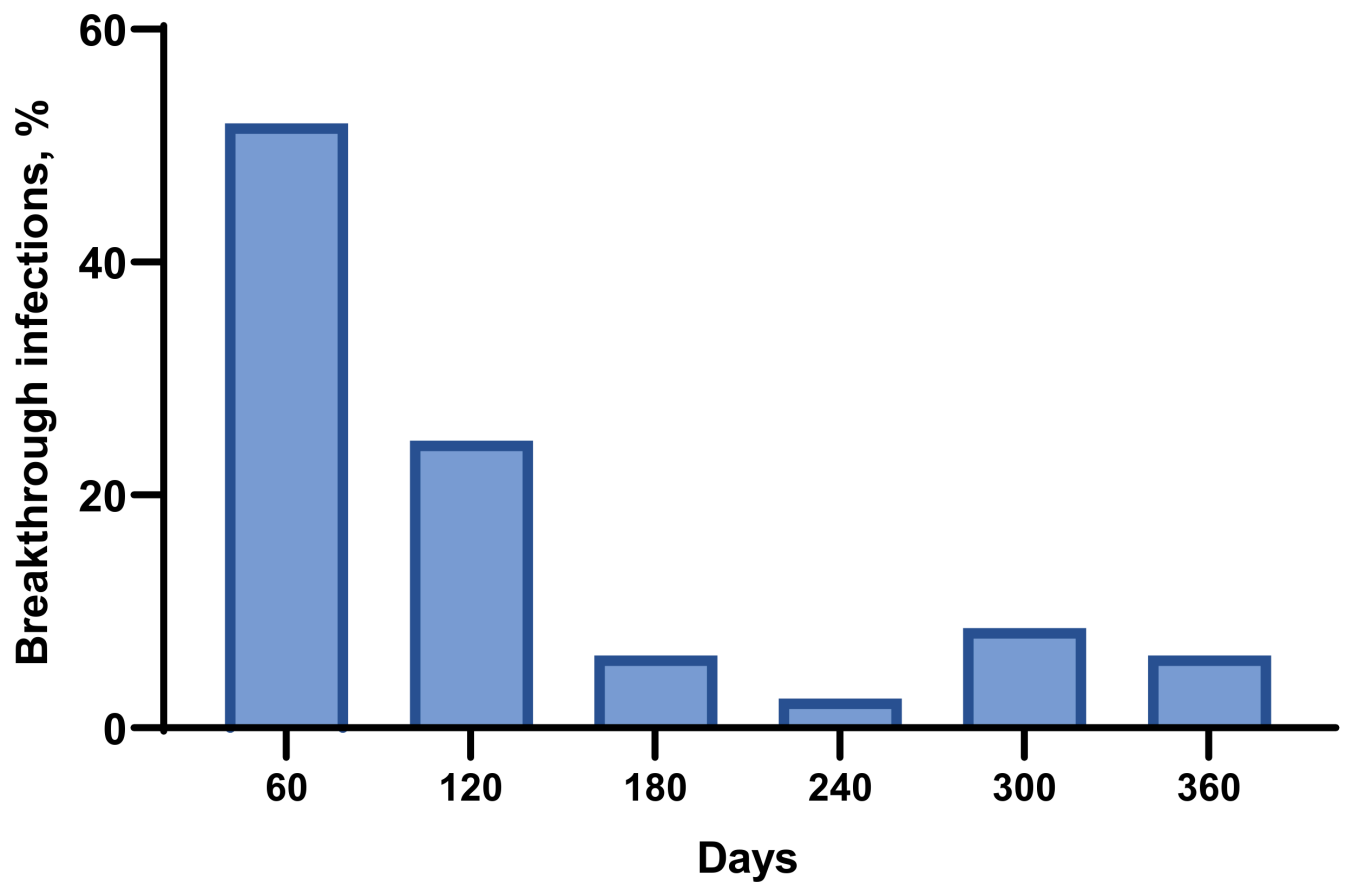
Nirsevimab-to-hospitalisation interval.

Among infants with breakthrough RSV infection, the interval between nirsevimab administration and hospitalisation ranged from 8 to 359 days (median 53.5 days, IQR 85 days). The distribution was not uniform, showing an early peak in breakthrough infections, a decline at intermediate intervals, and a secondary increase beyond 240 days after immunisation (Figure 1).

Comparing breakthrough infections with our cohort of patients hospitalised for RSV bronchiolitis who had not received immunisation with nirsevimab, we found that immunised patients were significantly more represented in the 9–12-month age group than the unimmunised cohort (Table 2). Of the 13 patients immunised with nirsevimab who were aged between nine and twelve months, only one had an underlying chronic condition, and none were born prematurely. The median distance from nirsevimab administration to admission was 291 days (IQR 110 days), and 76.9% of those patients were immunised at birth.

In multivariable logistic regression adjusted for age, prematurity, and comorbidities, nirsevimab immunisation was independently associated with significantly lower odds of requiring supplemental oxygen (aOR 0.33, 95% CI 0.19–0.57, *p* < 0.001). In a sensitivity analysis additionally adjusted for epidemic season, the association between nirsevimab and oxygen requirement was attenuated and no longer statistically significant (aOR 0.54, 95% CI 0.23–1.25, *p* = 0.150), with an unchanged direction of effect and a wider confidence interval (Table S3).

**Figure 2:**
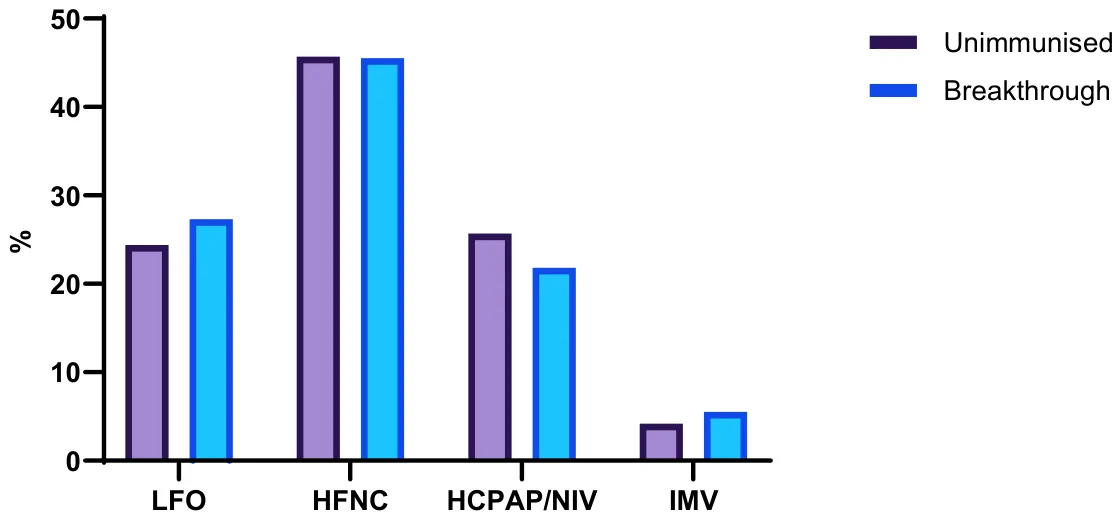
Maximum respiratory support required in the unimmunised and breakthrough cohort.

However, once the need for oxygen therapy emerged, the requirements of maximum ventilatory support were found to be comparable between the two cohorts (see Figure 2). Among infants who needed IMV, in the unimmunised cohort 3 patients (10.3%) had comorbidities and 5 (17.2%) were born prematurely. Notably, none of the three patients in the breakthrough group had comorbidities or were born prematurely.

Among infants who required oxygen therapy (n = 439; 1 excluded due to missing nirsevimab data), in multivariable logistic regression adjusted for age, prematurity and comorbidities, nirsevimab immunisation was not significantly associated with the need for advanced ventilatory support, defined as the need for HcPAP/NIV or IMV (aOR 0.92, 95% CI 0.48–1.76, *p* = 0.789). Older age was the only significant predictor of reduced odds of advanced ventilatory support (aOR 0.91 per month, 95% CI 0.833–0.989, *p* = 0.027).

Nirsevimab immunisation was not associated with advanced ventilatory support in the season-adjusted model (aOR 1.57, 95% CI 0.54–4.59, *p* = 0.405, Table S3).

### 3.3. Viral Coinfections

The coinfection status of six infants tested with RSV-specific PCR only could not be assessed, and these infants were excluded from coinfection-related analyses. Of the remaining 529 children, viral coinfection was present in 235 patients (44.4%). When stratified by nirsevimab status, viral coinfection was detected in 44.1% of unimmunised children and 45.7% of immunised children. As a sensitivity analysis, the multivariable models for oxygen requirement and advanced ventilatory support were repeated for the remaining patients, with the addition of viral coinfection as a covariate. The associations between nirsevimab immunisation and oxygen requirement (aOR 0.32, 95% CI 0.19–0.57, *p* < 0.001) and advanced ventilatory support among infants requiring oxygen (aOR 0.92, 95% CI 0.48–1.76, *p* = 0.793) were similar to those observed in the main models. Age was an independent predictor of the need for advanced ventilatory support (aOR 0.90 per month, 95% CI 0.83–0.99, *p* = 0.022).

## 4. Discussion

In this study, which was conducted across three epidemic seasons in a third-level hospital, we observed a significant reduction in RSV-related LRTI during the first year of life after the nationwide implementation of the nirsevimab immunisation programme.

A consistent drop in admissions was observed, with hospitalisations during the 2025–2026 epidemic season decreasing by 69.7% compared with the pre-nirsevimab era. However, the number of children that remained vulnerable to RSV during the most recent epidemic season is not negligible and warrants further investigation.

Patients who developed a breakthrough RSV infection in our study exhibited a higher prevalence of prematurity and underlying comorbidities compared with unimmunised patients. This finding is consistent with recent real-world evidence [26,30,37], and can be interpreted in two non-mutually exclusive ways. Firstly, it may reflect a shift in the residual risk profile of RSV infection, as nirsevimab substantially reduces the disease burden among otherwise healthy infants, thereby concentrating breakthrough cases among those with pre-existing vulnerabilities. Secondly, it may reflect selection bias arising from the preferential uptake of immunoprophylaxis among infants who are perceived to be at a higher clinical risk. The latter interpretation is consistent with the findings of a recent living systematic review and meta-analysis by Lee et al. [14], who reported that the presence of comorbidities and preterm birth did not significantly affect the overall effectiveness of nirsevimab.

Interestingly, patients with a breakthrough RSV infection tended to be more represented in the 9–12-month age group. This subgroup was largely healthy at baseline, and the median time since nirsevimab administration was also 291 days. This finding may be compatible with waning antibody-mediated protection; however, this interpretation remains hypothesis-generating, since the analysis is based only on breakthrough cases and does not account for the number of immunised infants remaining at risk or the corresponding person-time at each post-administration interval [38,39]. Prospective studies incorporating the at-risk population are needed to confirm this hypothesis. Nonetheless, these findings raise questions about the timing of nirsevimab administration in relation to the RSV season, as infants who are immunised in the mid-epidemic season may enter the subsequent season with a longer interval since prophylaxis, potentially during a period of intense viral circulation.

Nirsevimab has been shown to clearly reduce both the absolute number of hospital admissions and the need for oxygen therapy during hospitalisation for RSV bronchiolitis [20,24,40,41]. Our findings are in line with this protective effect, as immunised infants showed significantly lower odds of requiring supplemental oxygen (aOR 0.33, 95% CI 0.19–0.57, *p* < 0.001). However, this association should be interpreted with caution. Epidemic season is closely related to nirsevimab availability in our setting: no infant was immunised in 2023–2024, the product became available only from December 2024 and mainly for newborns in 2024–2025 [16], and it was widely used only in 2025–2026. When season was added to the model, the estimate was attenuated and imprecise (aOR 0.54, 95% CI 0.23–1.25), as it relied only on within-season comparisons in the last two seasons, with a consequent loss of effective sample size. We therefore cannot rule out that part of the observed association reflects temporal differences between seasons rather than the effect of nirsevimab alone. With regard to the level of respiratory support required, once the need for oxygen therapy emerged, the maximum level of ventilatory support was comparable between immunised and unimmunised infants, and immunisation status was not independently associated with the need for advanced respiratory support in the multivariable analysis. The only variable independently associated with reduced odds of advanced ventilatory support was older age, consistent with the greater susceptibility of younger infants to severe RSV lower respiratory tract disease [35,36]. Some recent studies similarly suggest that the clearest benefit of nirsevimab lies in preventing hospitalisation and oxygen-requiring disease overall, whereas evidence is less consistent for differences in escalation among infants who are already hospitalized with more severe illness [24,26,29,40,41,42]. This dissociation between the effects on oxygen requirements and ventilatory support may reflect the mechanism of action of nirsevimab, which targets the prefusion RSV F protein [43]. By primarily blocking viral entry and fusion through direct neutralisation, rather than reversing established inflammatory lung injury, nirsevimab is most plausibly expected to prevent infection or blunt early disease severity rather than modifying the course of severe infection once fully established [43,44]. However, this biological perspective should be interpreted with caution, as lower or less stable estimates for intensive care admission and advanced ventilatory support may reflect both the rarity of these endpoints and the fact that most studies evaluate prevention of severe outcomes at the population level rather than stage-specific deterioration after admission [37,45]. Further large-scale, prospective studies, specifically designed to evaluate the progression to critical illness and advanced respiratory support using clearly defined severity-escalation endpoints, are required to distinguish between these alternative explanations.

Further consideration from our cohort arises from the clinical profile of infants that required IMV. In the unimmunised cohort, a proportion of infants who progressed to IMV had underlying comorbidities (10.3%) or were born prematurely (17.2%). In contrast, none of the three infants who required IMV in the breakthrough group had comorbidities or a history of prematurity. Although the small number of cases precludes any conclusions, this pattern suggests that, even in the era of nirsevimab immunoprophylaxis, severe RSV breakthrough cases still occur and are not confined to traditionally high-risk infants [25,30]. This underlines the importance of maintaining a high level of clinical vigilance and close monitoring of all young infants hospitalised with bronchiolitis, regardless of their immunisation status or the presence of traditional risk factors. Although this observation remains purely descriptive, considering the small number of cases, it is consistent with the broader rationale for universal prophylaxis of all healthy infants, supported by larger studies [21,24,38], as severe disease can occur even in the absence of recognised risk factors. Further prospective studies investigating variability in neutralising antibody titres and the individual immune response are needed to elucidate the immunopathogenic mechanisms underlying severe disease despite adequate immunoprophylaxis.

This study has limitations to be acknowledged: firstly, it is a single-centre study, which may limit the generalisability of the results to other settings. Secondly, severe outcomes such as the need for invasive mechanical ventilation were uncommon, limiting statistical precision and making subgroup comparisons vulnerable to instability. Thirdly, immunisation status was closely related to epidemic season, since nirsevimab exposure was possible only from the 2024–2025 season. In a sensitivity analysis including season, the association with oxygen requirement was attenuated and no longer statistically significant, and residual confounding by seasonal differences in RSV circulation, healthcare access and respiratory-support practices cannot be excluded. Finally, being a retrospective study, it relies on data collected from clinical records, which may be incomplete or inconsistently recorded.

This study also has several strengths: it was conducted in a third-level hospital and included a substantial cohort of 536 RSV-positive infants, out of 952 infants hospitalised for bronchiolitis overall. Notably, this sample size was achieved in a period characterised by a progressive decline in RSV-related hospitalisations following the introduction of nirsevimab immunoprophylaxis, reflecting the real-world effectiveness of the immunisation programme; in this context, our cohort represents a comparatively large and clinically meaningful sample for assessing outcomes among both immunised and unimmunised infants. Furthermore, 98.9% of patients admitted with bronchiolitis underwent testing with an extended multiplex PCR respiratory panel at admission, allowing for accurate and systematic aetiological characterisation and minimising the risk of misclassification or of missing RSV-positive patients. This comprehensive virological testing strategy ensured that the study population was well characterised and that the observed associations could be more confidently attributed to RSV-related disease.

## 5. Conclusions

Overall, our findings are consistent with the substantial real-world impact of the nirsevimab immunisation programme, with a marked reduction in RSV-related hospitalisations and in the need for supplemental oxygen therapy among infants admitted with RSV bronchiolitis in the main model; this association was attenuated in a sensitivity analysis adjusted for epidemic season, a model with limited precision given the close relationship between season and nirsevimab availability. Once hospitalisation and oxygen requirement had occurred, immunisation status did not independently modify the need for advanced ventilatory support. This observation should be interpreted with caution, given the limited number of severe events in our cohort, and warrants further investigation in larger studies. Breakthrough infections, although less frequent, were not confined to infants with traditional risk factors, such as prematurity or comorbidities. These findings emphasise the importance of maintaining a high level of clinical vigilance and close respiratory monitoring of all young infants hospitalised with bronchiolitis, regardless of immunisation status or recognised risk factors, and further corroborate the growing body of evidence supporting a universal, rather than risk-based, approach to nirsevimab prophylaxis.

## Figures and Tables

**Table 1:** Demographic and clinical characteristics of patients admitted with RSV bronchiolitis, by epidemic season.

	2023–2024 *N = 267*	2024–2025 *N = 188*	2025–2026 *N = 81*	*p*
M/F	140/127	108/80	44/37	0.572
Age				<0.001
<3 months	191 (71.5)	132 (70.2)	40 (49.4)	
3–6 months	50 (18.7)	28 (14.9)	15 (18.5)	
6–9 months	13 (4.9)	19 (10.1)	10 (12.3)	
9–12 months	13 (4.9)	9 (4.8)	16 (19.8)	
Preterm birth	15 (5.6)	12 (6.4)	8 (9.9)	0.408
Weight at birth, grams	3300 (595)	3250 (530)	3240 (645)	0.348
Comorbidity	8 (3.0)	8 (4.3)	7 (8.6)	0.090
Nirsevimab	-	18 (9.6)	63 (77.8)	<0.001
Length of stay, days	6 (4.0)	5 (4.0)	5 (3.5)	0.014 *
O_2_ therapy	231 (86.5)	154 (81.9)	55 (67.9)	0.001
Max respiratory support				0.268
LFO	64 (27.7)	29 (18.8)	16 (29.1)	
HFNC	98 (42.4)	76 (49.4)	27 (49.1)	
HcPAP/NIV	58 (25.1)	44 (28.6)	9 (16.4)	
IMV	11 (4.8)	5 (3.2)	3 (5.5)	

* Pairwise post-hoc comparisons: 2023–2024 vs. 2025–2026, *p* = 0.011.

**Table 2:** Demographic and clinical characteristics of the unimmunised cohort and the breakthrough cohort.

	Unimmunised Cohort *N = 454*	Breakthrough Infections *N = 81*	*p*
M/F	249/205	43/38	0.770
Age			0.006 *
<3 months	314 (69.2)	49 (60.5)	
3–6 months	80 (17.6)	13 (16.0)	
6–9 months	36 (7.9)	6 (7.4)	
9–12 months	24 (5.3)	13 (16.0)	
Preterm birth	25 (5.5)	10 (12.3)	0.023
Weight at birth, grams	3290 (630)	3220 (620)	0.302
Comorbidity	16 (3.5)	7 (8.6)	0.066
Length of stay, days	5.0 (4.0)	5 (4.0)	0.013
O_2_ therapy	385 (84.8)	55 (67.9)	<0.001
Max respiratory support			0.888
LFO	94 (24.4)	15 (27.3)	
HFNC	176 (45.7)	25 (45.5)	
HcPAP	99 (25.7)	12 (21.8)	
IMV	16 (4.2)	3 (5.5)	

* Post hoc comparison Bonferroni-corrected: 9–12 months, breakthrough infections vs. unimmunised cohort, *p* = 0.002; no significant differences in the other age categories.

## Data Availability

The anonymised dataset is not publicly available due to institutional data protection policies regarding paediatric clinical data. Access may be granted upon reasonable request to the corresponding author, subject to approval by the Ethics Committee of Bambino Gesù Children’s Hospital and execution of a data-sharing agreement, for research purposes consistent with the original ethical approval.

## Supplementary Materials

The following supporting information can be downloaded at: https://www.mdpi.com/article/10.3390/v18101107/s1, Table S1. Demographic and clinical characteristics of patients that required oxygen therapy in the unimmunised cohort and the breakthrough cohort. Table S2. Institutional criteria for initiating respiratory support. Table S3. Multivariable logistic regression model including epidemic season as an additional covariate.

## Abbreviations

The following abbreviations are used in this manuscript:

aOR	Adjusted odds ratio
CI	Confidence interval
HcPAP	Helmet continuous-positive airway pressure
HFNC	High-flow nasal cannula
ICU	Intensive care unit
IQR	Interquartile range
IMV	Invasive mechanical ventilation
LFO	Low-flow oxygen
LRTI	Lower respiratory tract infections
N	number
NIV	Non-invasive ventilation
O2	Oxygen
PCR	Polymerase chain reaction
RSV	Respiratory syncytial virus
