# Supplementary material for: Clinical Profile of RSV Breakthrough Infections After Nirsevimab Introduction
Source: Viruses. 2026 Oct 7;18(10):1107. doi: 10.3390/v18101107 (PMC13709675; doi:10.3390/v18101107)
Supplement: Supplementary file 1 [file viruses-18-01107-s001.zip › viruses-4576532-supplementary.pdf]

Table S1. Demographic and clinical characteristics of patients that required oxygen therapy in the unimmunised cohort and the breakthrough cohort.

	**Unimmunised cohort** *N=385*	**Breakthrough infections** *N=55*	**p**
M/F	217/168	30/25	0.799
Age			0.014
<3 months	269 (69.9)	29 (52.7)	
3-6 months	67 (17.4)	12 (21.8)	
6-9 months	27 (7.0)	5 (9.1)	
9-12 months	22 (5.7)	9 (16.4)	
Preterm birth	23 (6.0)	8 (14.5)	0.042
Weight at birth	3330 (612)	3215 (670)	0.369
Comorbidity	15 (3.9)	7 (12.7)	0.012
Length of stay, days	6.0 (5.0)	5 (4.0)	0.203

Table S2. Institutional criteria for initiating respiratory support.

**Level of support**	**Criteria for initiation**
Low-flow oxygen (LFO)	SpO2 <90–92%, with a target SpO2 ≥93–94%
High-flow nasal cannula (HFNC)	PaCO2 >55 mmHg, or persistent SpO2 <90% (in infants without chronic cardiac or respiratory disease) with increased work of breathing and respiratory rate >50/min
Helmet CPAP/non-invasive ventilation (HcPAP/NIV)	Signs of respiratory fatigue (respiratory rate >50/min), accessory muscle use and paradoxical breathing, or hypoxaemia despite high FiO2 (P/F ratio ≤250) with pH <7.30 and PaCO2 >45 mmHg
Invasive mechanical ventilation (IMV)	Based on clinical judgement, in the presence of respiratory failure, apnoea with desaturation, or shock; no protocol-defined numerical threshold

Table S3. Multivariable logistic regression model including epidemic season as an additional covariate.

	Oxygen requirement N=532		Advanced ventilatory support N=439	
	aOR (95% CI)	p	aOR (95% CI)	p
Nirsevimab (yes vs no)	0.54 (0.23–1.25)	0.150	1.57 (0.54-4.59)	0.405
Age, months	1.03 (0.94–1.12)	0.544	0.92 (0.84-0.99)	0.048
Preterm birth	1.88 (0.62–5.71)	0.265	1.30 (0.59-2.86)	0.521
Comorbidity	5.79 (0.75–44.99)	0.093	1.64 (0.64-4.21)	0.306
Season 2024–2025 vs 2023–2024	0.70 (0.41–1.19)	0.186	1.07 (0.68-1.68)	0.774
Season 2025–2026 vs 2023–2024	0.46 (0.18–1.19)	0.108	0.49 (0.15-1.58)	0.231
Season (overall)		0.213		0.388
